# Outcomes of Early vs. Late Surgical Intervention in Children With Undescended Testis

**DOI:** 10.7759/cureus.56430

**Published:** 2024-03-19

**Authors:** Lama AlSahli, Abdulaziz Alabdulsalam, Arwa Mahfouz, Mohammad Alnamshan

**Affiliations:** 1 College of Medicine, King Saud Bin Abdulaziz University for Health Sciences, Riyadh, SAU; 2 Pediatric Surgery, King Abdulaziz Medical City Riyadh, Riyadh, SAU

**Keywords:** outcome, testicular size, infertility, orchidopexy, cryptorchidism, undescended testis

## Abstract

Background

Undescended testis (UDT) is one of the most common urogenital abnormalities. International guidelines recommend performing orchidopexy no later than 18 months to decrease the risk of complications associated with UDT such as infertility and testicular malignancy. The aim of the study is to evaluate the outcomes of early versus late surgical intervention of UDT and to assess if the optimal age of orchidopexy is met.

Methods

This is a retrospective cross-sectional study that included 258 pediatric patients' testes with no prior UDT intervention from January 2016 to December 2020. A chart review was used to collect the patients’ data. Children included were categorized into two groups based on their age at the time of surgery (group A ≤ 18 months and group B > 18 months). Statistical differences were explored using Pearson's chi-squared test or Fisher's exact test for categorical variables or a Wilcoxon rank sum test for numerical variables. A p-value of <0.05 indicated statistical significance.

Results

The median age at the presentation among the overall cohort was 14 months. The median age at the presentation for group A was six months and group B was 35 months. Group A included 109 children and group B included 149 children. At the time of the surgery, the median age of patients was 23 months. The median age at orchidopexy for group A was 12 months and the median age for group B was 38 months. The time between diagnosis and surgery was significantly shorter among older children with a median duration of one month versus a median of five months among patients in the ≤18 months group (p = 0.003). The follow-up interval was at three and 12 months. The change in testicular size before and after surgeries was statistically significant, as most small testicles before surgeries had become normal in size after surgeries among the overall cohort (76.6%), patients aged ≤18 months (72.4%), and those aged >18 months (79.2%) (p < 0.001).

Conclusion

Most of the patients included in this study did not undergo orchidopexy at the optimal age recommended by the international guidelines. However, there was a statistically significant improvement in testicular size following orchidopexy in children with small UDT regardless of age at the time of surgery.

## Introduction

Undescended testis (UDT) or cryptorchidism refers to the absence of one or both testes from the scrotum at the time of birth [1]. It is one of the most common urogenital abnormalities in males with a prevalence of 2%-4% reaching as high as 30-45% in preterm infants [2]. This condition results due to failure of the normal testicular descent and fixation to the scrotal sac. Despite extensive research efforts to identify the exact cause of UDT, it remains unknown. Several risk factors for the development of UDT have been identified. Prematurity and low birth weight are considered major risk factors that increase the likelihood of developing the condition. In full-term infants, the condition has been attributed to genetic, maternal, or environmental factors which are thought to influence the normal testicular development and process of descent [2].

The testes may be absent or instead found along the usual route of descent with either an intra-abdominal, inguinal, suprascrotal, or a high scrotal position [3]. In boys with an impalpable testis, it may be attributed to testicular atrophy, ectopic testis, or an intra-abdominal or inguinal location [3]. In most of the cases, spontaneous descent of the testis occurs during the first three months of life. If the testis remains undescended by six months of age, it is less likely to reach its proper position, and surgical intervention is deemed necessary. The proper development and growth of the testis are reliant on its fixation in the scrotal sac by the time of birth [2,3]. In the cases of UDT, studies investigating the timing of surgical fixation have concluded that early intervention leads to improved testicular volume at the time of follow-up [4,5].

Several studies have revealed that UDT is associated with the development of complications in adulthood [6,7]. A majorly reported consequence of a persistent UDT is severely impaired spermatogenesis. If UDT is left untreated, this could lead to compromised fertility in adulthood. The risk of infertility is greater in men born with bilateral cryptorchidism [6]. Another reported complication is an increased risk of testicular malignancy [7]. The increasing knowledge regarding the different consequences of untreated UDT has driven progressive adjustments to the recommended age of surgical intervention. The main goals of treating UDT are to improve testicular growth, preserve fertility, decrease the risk of malignancy [8], and enable the patient to examine the testis to detect any abnormal masses. To prevent the associated consequences of UDT, the most recently published recommendation is to perform orchidopexy between six and 12 months, and no later than 18 months [8]. Nevertheless, the optimal time frame for orchidopexy remains unmet and delayed surgical correction has been reported by multiple studies [9,10].

In Saudi Arabia, the overall prevalence of UDT is unknown. A few studies have looked into the age of presentation and surgical intervention in boys with UDT [11,12]. Since local studies have revealed late presentation, this study aims to determine the age of presentation of UDT to our institution and to evaluate if the optimal age of orchidopexy is met. Another important aim is to assess the outcomes of early versus late surgical intervention through assessment of testicular volume.

## Materials and methods

This research was conducted at King Abdullah Specialized Children’s Hospital (KASCH), Riyadh, Saudi Arabia. It is the only medical institute to be exclusively specialized in the care of children in Saudi Arabia. Since its establishment in 2015, KASCH has continued expanding and providing a wide range of advanced services and treatment modalities for all patients in need. KASCH aims to provide comprehensive care that is unique to the needs of each child. Through the use of advanced technology, KASCH is dedicated to achieving optimum health outcomes for children and their families by adopting a multidisciplinary approach to childcare. The hospital is equipped with 14 operating rooms and two interventional radiology rooms for emergency and elective surgeries.

This study was a retrospective cross-sectional study. Files of patients meeting the inclusion criteria were reviewed on BESTCare.

Inclusion criteria were all pediatric patients with UDT who underwent orchidopexy from January 2016 to December 2020, had no previous intervention to the UDT, and patients who were followed postoperatively for three and 12 months. Exclusion criteria were previous surgical intervention to UDT, vanishing (atrophied) testes, patients with a past medical history, patients with a past surgical history, patients who have any known allergies, patients who lost follow-up after orchidopexy, had incomplete medical records, preterm births, or older than 13 years of age.

The optimal sample size using the Raosoft Sample Calculator is 258 testes in a total of 217 patients assuming the margin of error to be 5%, confidence level 95%, and an estimated population of 776, which were on the list of orchidopexy in general pediatric surgery and pediatric urology surgery lists from January 2016 to December 2020.

The testicular size is estimated by the examining physician's subjective measurement. No specific tool was used to measure the testicular size.

Statistical analysis was carried out using RStudio (R version 4.1.1; R Foundation for Statistical Computing, Vienna, Austria). Categorical variables were presented as frequencies and percentages, whereas numerical variables were expressed as median and interquartile range (IQR). Children under study were categorized into two groups based on their age at the time of surgery (group A ≤18 months and group B >18 months). Statistical differences were explored using Pearson's chi-squared test or Fisher's exact test for categorical variables or Mann-Whitney U test for numerical variables. These differences were explored based on children’s age (group A ≤18 months or group B >18 months) and the incidence of complications (No or Yes). Temporal changes in the size of testicles before and after surgeries were assessed using McNemar's chi-squared test with continuity correction. A p-value of <0.05 indicated statistical significance.

## Results

We included the data of 258 pediatric patients' testes under pediatric surgery and pediatric urology in the current study. The median (IQR) age at presentation was 14.0 months (6.0, 37.0). The majority of testicles were palpable (88.8%) and were located in the inguinal region (82.6%). More than two-thirds of children had unilateral UDT (81.11%), and the affected testicles were on the left side among 50.4% of children (Table 1).

**Table 1:** Demographic and clinical parameters at presentation among the overall cohort and categorized by the age at surgery. *The p-value is based on Pearson's chi-squared test or Fisher's exact test for categorical variables or Mann-Whitney U test for numerical variables. IQR: interquartile range.

Parameter	Category	Overall, N = 258	Age at surgery		
			≤18 months, N = 109	>18 months, N = 149	p-Value*
Age at presentation (months)	Median (IQR)	14.0 (6.0, 37.0)	6.0 (5.0, 10.0)	35.0 (19.0, 60.0)	<0.001
	Range	1-120	1-17	2-120	
Age at surgery (months)	Median (IQR)	23.0 (13.0, 40.0)	12.0 (9.0, 14.0)	38.0 (24.0, 60.0)	<0.001
	Range	6-120	6-18	19-120	
Time to surgery (months)	Median (IQR)	3.0 (0.0, 7.0)	5.0 (2.0, 7.0)	1.0 (0.0, 8.0)	0.003
	Range	0-54	0-13	0-54	
Palpation upon clinical exam	Palpable	229 (88.8%)	94 (86.2%)	135 (90.6%)	0.273
	Impalpable	29 (11.2%)	15 (13.8%)	14 (9.4%)	
Laterality	Unilateral	176 (81.11%)	79 (84.04%)	97 (78.86%)	0.209
	Bilateral	41 (18.89%)	15 (15.96%)	26 (21.14%)	
Side	Left	130 (50.4%)	56 (51.4%)	74 (49.7%)	0.786
	Right	128 (49.6%)	53 (48.6%)	75 (50.3%)	
Location	Intra-abdominal	7 (2.7%)	4 (3.7%)	3 (2.0%)	0.093
	Inguinal	213 (82.6%)	94 (86.2%)	119 (79.9%)	
	High scrotal	15 (5.8%)	2 (1.8%)	13 (8.7%)	
	Ectopic	23 (8.9%)	9 (8.3%)	14 (9.4%)	

At the time of the surgery, the median age (IQR) of patients was 23.0 months (13.0, 40.0), and it ranged from six to 120 months.

In general, we divided the patients into two groups. Group A included 109 patients aged ≤18 months (42.2%) and group B included 149 patients aged 18 months or more (57.8%). Children in group B were significantly older at presentation (median = 35.0 months, IQR = 19.0-60.0 vs median = 6.0 months, IQR = 5.0-10.0 among patients in group A patients, p < 0.001).

Time to surgery was significantly shorter among older children (median = 1.0 months, IQR = 0.0-8.0 vs median = 5.0 months, IQR = 2.0-7.0 among patients in group A, p = 0.003). Statistical differences were not found between the children’s groups in terms of other clinical parameters at presentation (Table 1).

Surgery-related characteristics

In the majority of cases, orchidopexy was performed using the open approach (89.1%) and 4.7% using the laparoscopic approach, whereas 6.2% of them were converted from the laparoscopic to open technique if the internal inguinal ring was found to be closed on exploratory laparoscopy indicating that the testis has passed the internal ring. Testicular size changed from the pre- to postoperative period among 25.6% of patients, where the size reduced among 3.9% and increased among 21.7% of children. The postoperative period is identified as the second and third follow-up visits at the clinic which were at 12 to 18 months after the surgery. Complications occurred in three children (1.2%); all of these complications occurred in group 2 patients. However, there were no significant changes in the rates of complications, types of surgery, and testicular size between the study groups (Table 2).

**Table 2:** Surgery-related characteristics among the overall cohort and categorized by the age at surgery. *The p-value is based on Pearson's chi-squared test or Fisher's exact test.

Parameter	Category	Overall, N = 258	Age at surgery		
			≤18 months, N = 109	>18 months, N = 149	p-Value*
Type of surgery	Open	230 (89.1%)	95 (87.2%)	135 (90.6%)	0.206
	Laparoscopic	12 (4.7%)	8 (7.3%)	4 (2.7%)	
	Laparoscopic to open	16 (6.2%)	6 (5.5%)	10 (6.7%)	
Pre-op size vs post-op	Smaller	10 (3.9%)	4 (3.7%)	6 (4.0%)	0.345
	The same	192 (74.4%)	86 (78.9%)	106 (71.1%)	
	Improved	56 (21.7%)	19 (17.4%)	37 (24.8%)	
Complications	Yes	3 (1.2%)	0 (0.0%)	3 (2.0%)	0.265

Changes in testicular size

Generally, 77 children had small testicles before the operation. Of them, 59 testicles became the size of the contralateral normally descended testis after the procedure (76.6%). The change in testicular size before and after surgeries was statistically significant, where most small testicles before surgeries had become normal in size compared to the contralateral normally descended testis after surgeries among the overall cohort (76.6%, p of change < 0.001), patients in group A (72.4%, p of change = 0.001) and those in group B (79.2%, p of change < 0.001, Table 3).

**Table 3:** The status of testicular size before and after 12-18 months of the surgeries. *The p-values are based on McNemar's chi-squared test.

Parameter	Category	Overall, N = 258	Size before		
			Small	Good	p-Value*
Size after surgery (overall cohort)	Small	27 (10.5%)	18 (23.4%)	9 (5.0%)	<0.001
	Increased	231 (89.5%)	59 (76.6%)	172 (95.0%)	
Size after surgery in group A (≤18 months, n = 109)	Small	12 (11.0%)	8 (27.6%)	4 (5.0%)	0.001
	Increased	97 (89.0%)	21 (72.4%)	76 (95.0%)	
Size after surgery in group B (>18 months, n = 149)	Small	15 (10.1%)	10 (20.8%)	5 (5.0%)	<0.001
	Increased	134 (89.9%)	38 (79.2%)	96 (95.0%)	

Factors associated with complications

Results of the association analysis showed that the incidence of complications was not correlated with any of the demographic, clinical, and surgery-related characteristics.

## Discussion

In the literature, multiple studies have assessed the age of presentation in boys with UDT and the influencing factors of delayed presentation [12,13]. Unfortunately, both international and local studies have shown a deviation from the international guidelines' recommendations. The reported ages at presentation and surgery, and the mean waiting time for orchidopexy were variable across the literature.

Age at presentation 

In Saudi Arabia, Alsowayan et al. retrospectively reviewed 195 cases diagnosed with UDT and found that the mean age of presentation was 13.7 months, which is consistent with the findings of the present study [11]. The median age of presentation in our cohort of patients was 14.0 months (6.0, 37.0). Another local study conducted by Alnoaiji et al. included 175 patients and found that the median age at UDT diagnosis was 12.0 months (3, 24) [14].

Late presentation of children with UDT may be attributed to delayed identification and referral by the physician at the primary healthcare clinic or during the scheduled vaccination appointments. Also, parental misconceptions regarding the risks of surgery might be another possible cause.

Testicular position

Regarding UDT position, a study done in Taiwan by Chi-Shin Tseng et al. stated that the inguinal canal is the major site of the UDT (75.2%) [15]. Another study conducted by Chi-Shin Tseng et al. involving 182 patients revealed that 134 had unilateral UDT, and most of the testes were found inguinal in position intraoperatively (84.3%) [16]. Locally, Alnoaiji et al. discovered that 80.6% of the UDT cases were located in the inguinal canal [14]. In the current study, the testicular position was assessed at presentation at the clinic and was confirmed with examination under anesthesia. Interestingly, among our cohort of patients, the majority were also found in the inguinal canal (82.6%) both preoperatively and intraoperatively.

Laterality

Internationally, a study conducted in India by Mallikarjuna M, et al. concluded that most of the UDT cases were unilateral and involved the right testis (60%) [17]. Another study done in Taiwan revealed that 73.6% had unilateral UDT and mostly involved the left side (39.5%) [15]. Another international study by Riaz Ahmed et al. which was done in Karachi, Pakistan, found that among 91 patients involved in the study, 47 patients (51.6%) had unilateral left UDT [18]. Moreover, a local study by Alnoaiji et al. concluded that most of the UDT cases in their study were unilateral left (42.9%) [14]. Similarly, the present study found that most of the children had unilateral UDT (81.11%), and the affected side was the left testis (50.4%).

Age at surgery

Internationally, a recent study conducted in the United States revealed that 68% of boys with UDT underwent orchidopexy after the age of two years [19]. Another study examining the age of orchidopexy in Victoria, Australia, reported that up to 55% of boys were older than five years at the time of surgery [20]. In contrast, another Australian study by Schneuer et al. reported the median age of orchidopexy to be 16.6 months [21]. More favorable results that are in line with the international guidelines were reported by Alsaywid in Sydney, Australia, as almost three-quarters of patients were less than two years of age with the median age at surgery being 11 months [22].

Locally, Alsowayan et al. [11] reported a mean age of 25 months at orchidopexy. Similarly, the median age in the present study was 23.0 months at the time of surgery.

Alhazmi et al. [12] reported a far-from-ideal age at surgery with a median age of 43.7 months, which had been attributed to a delayed referral time of a median of 21.3 months. In 2009, Neel reviewed 345 cases of UDT at two centers in Riyadh, Saudi Arabia [23]. In his study, the mean age of orchidopexy was 54.8 months, and only 29.5% were operated on before their first birthday. However, in Neel’s study, there was no mention of the reason for delayed management [23].

Waiting time for the surgery

In the present study, the wait time for surgery in the older children aged >18 months was significantly shorter (p < 0.001) than the ≤18 months group, with a median of one month. These findings reflect a priority of fixation in older children at our institution. In Saudi Arabia, the median waiting time for elective surgery has been assessed by multiple studies. Alsowayan et al. reported an overall median waiting time of 4.8 months (<1 day to 49.4 months) for elective surgery [11]. In addition, Alnoaiji et al. reported a median duration between diagnosis and surgery of eight months [14]. Furthermore, a longer waiting time for surgery was reported by Alhazmi et al. Their results showed that the median period between diagnosis and surgical fixation of UDT was 15.2 months [12].

Testicular size before and after the surgery

Chi-Shin Tseng et al. reported that in all types of UDT, namely suprascrotal, inguinal, and above-inguinal UDTs, patients had a smaller testis in comparison to the normally descended testis. Adding that the higher the position of the UDT, the smaller the testicular volume [15]. A local study by Alnoaiji et al. found that the majority of the UDT cases were average in size before orchidopexy [14]. Similarly, our study found that the majority of UDT cases were normal in size at the initial visit.

Alnoaiji et al. found that there was no significant association between the time of orchidopexy and the improvement of the testis size, which is consistent with the findings of the present study [14]. The current study found that there was no statistically significant change between preoperative and postoperative testicular size. However, there was a statistically significant (p < 0.001) improvement in testicular size after orchidopexy in patients who had small testis preoperatively.

In 2013 in Sweden, Claude Kollin et al. reported that children with UDT who underwent orchidopexy at nine months of age had larger testicular volume at five years of age during follow-up compared with those who underwent orchidopexy at the age of three years [24]. These findings are supported by a study by C. Kollin et al., which stated that at four years of follow-up, children operated on at the age of nine months had a larger testicular volume than those who were operated on at the age of three years excluding intra-abdominal testes [5].

Complications

In 175 orchidopexy surgeries, Alnoaiji et al. explored the occurrence of postoperative complications and found that only 4% developed postoperative complications [14]. Most of these complications were wound infections (1.7%). No associations were found between the timing of the surgery and the development or type of complications in their study. Similarly, only three complications (1.2%) occurred in the current study. These complications occurred in group B (>18 months) and were minimal wound dehiscence, mild serosanguineous discharge, and wound infection. Other orchidopexy complications reported by international studies were testicular atrophy and ascent [25,26].

Limitations

There are limitations to this study. A potential limitation is the subjective measurement of testicular size. The testicular size was estimated by palpation at the initial visit, intraoperatively, and at the follow-up visits by the physician. These measurements may be underestimated or overestimated by the examining physician and are subject to bias and errors. We recommend the use of an objective tool such as ultrasonography in future studies to accurately assess the change in testicular volume.

## Conclusions

Most of the patients included in this study did not undergo orchidopexy at the optimal age recommended by the international guidelines. However, there was a statistically significant improvement in testicular size following orchidopexy in children with small UDT regardless of age at surgery. UDT should be actively screened for since early intervention preserves testicular volume. Thus, the implementation of structured programs and targeted educational campaigns is necessary to raise awareness regarding the complications of delayed surgical fixation and to emphasize the importance of timely management. Additionally, the causes of late presentation and late surgical intervention, including missing diagnosis at birth, unnoticed UDT by the family, late clinic appointment availability, long follow-up intervals, and long surgery waiting lists, should be addressed to improve the outcomes of UDTs. Future research should focus on identifying specific strategies to reduce delay times and enhance health education initiatives, thereby ensuring timely and effective management of UDT in pediatric patients.
